# Rice Protein Exerts Anti-Inflammatory Effect in Growing and Adult Rats via Suppressing NF-κB Pathway

**DOI:** 10.3390/ijms20246164

**Published:** 2019-12-06

**Authors:** Zhengxuan Wang, Mingcai Liang, Hui Li, Liang Cai, Lin Yang

**Affiliations:** Department of Food Science and Engineering, School of Chemistry and Chemical Engineering, Harbin Institute of Technology, Harbin 150001, China; 18646199358@163.com (Z.W.); hitmcl@163.com (M.L.); huili_1024@163.com (H.L.); feilan1984@sina.com (L.C.)

**Keywords:** rice protein, anti-inflammatory activity, NF-κB, age, rats

## Abstract

To elucidate the effect of rice protein (RP) on the depression of inflammation, growing and adult rats were fed with caseins and RP for 2 weeks. Compared with casein, RP reduced hepatic accumulations of reactive oxygen species (ROS) and nitro oxide (NO), and plasma activities of alanine transaminase (ALT) and aspartate transaminase (AST) in growing and adult rats. Intake of RP led to increased mRNA levels, and protein expressions of phosphoinositide 3 kinase (PI3K), protein kinase B (Akt), nuclear factor-κB 1 (NF-αB1), reticuloendotheliosis viral oncogene homolog A (RelA), tumor necrotic factor α (TNF-α), interleukin-1β (IL-1β), interleukin-6 (IL-6), inducible nitric oxide synthase (iNOS), cyclooxygenase-2 (COX-2), and monocyte chemoattractant protein-1 (MCP-1) were decreased, whereas hepatic expressions of interleukin-10 (IL-10) and heme oxygenase 1 (HO-1) were increased by RP. The activation of NF-κB was suppressed by RP through upregulation of inhibitory κB α (IκBα), resulting in decreased translocation of nuclear factor-κB 1 (p50) and RelA (p65) to the nucleus in RP groups. The present study demonstrates that RP exerts an anti-inflammatory effect to inhibit ROS-derived inflammation through suppression of the NF-κB pathway in growing and adult rats. Results suggest that the anti-inflammatory capacity of RP is independent of age.

## 1. Introduction

Inflammation, an important response to infection and injury, is implicated in various diseases, e.g., metabolic syndrome, etc. [1]. Oxidative stress, which can be caused by the overproduction of reactive oxygen species (ROS), is one of the most potent inducers of inflammation [2]. Thus, the suppression of oxidative stress is suggested to be very useful for preventing inflammatory diseases.

As a major plant protein, rice protein (RP) has numerous physiological functions [3,4,5,6], including induction of the antioxidant response [7,8,9,10]. Consequently, rice protein can reduce oxidative stress by scavenging nitric oxide (NO) radicals, ROS (e.g., superoxide radical, hydrogen peroxide), etc. [11,12,13], which have been suggested as the major causes of inflammation. Thus, there is evidence supporting the notion that rice protein might exert an antioxidant capacity to prevent inflammation. However, until now, a comprehensive understanding of the link between the suppression of oxidative stress-induced inflammation with the intake of rice protein has not been elucidated. Particularly, although it has been demonstrated that rice protein hydrolysates can inhibit the inflammatory response in mouse leukemia cells of monocyte macrophage (RAW264.7) [14], the precise molecular mechanism and signal pathway by which rice protein prevents ROS-derived inflammation in growing and adult rats are not fully established.

Nuclear factor-κB (NF-κB) is a master regulator in the inflammatory process [1]. The NF-κB family consists of five proteins, NFκB1 (p105), NFκB2 (p100), reticuloendotheliosis viral oncogene homolog A (RelA, p65), reticuloendotheliosis viral oncogene homolog B (RelB), and c-reticuloendotheliosis viral oncogene homolog (c-Rel) [1,15]. In general, NF-κ exists as a heterodimeric complex of p50 and p65 subunits. Under physiological conditions, NF-κB is present in an inactive form in the cytoplasm, complexed to several NF-κB-inhibitors, which is called inhibitory κB (IκB), e.g., IκBα. As stimulation, IκB proteins are degraded, resulting in the NF-κB complex being translocated into the nucleus [1,15]. Upon activation of NF-κB, the transcription of a number of inflammatory genes can be activated, e.g., tumor necrotic factor alpha (*TNF-α*), interleukin-1β (*IL-1β*), interleukin-6 (*IL-6*), inducible nitric oxide synthase (*iNOS*), cyclooxygenase-2 (*COX-2*), monocyte chemoattractant protein-1 (*MCP-1*), etc. [1,16,17,18,19]. This is the basic functional mechanism through which the NF-κB transcription factor induces an inflammatory response. Thus, in order to elicit the anti-inflammatory mechanism exerted by rice protein, the impact of rice protein on the activation of NF-κB should be taken into account.

Age is a major factor in inflammation. NF-κB activity has been shown to be upregulated with age, suggesting that NF-κB signaling is a culprit of inflamm-aging [15,20]. Moreover, NF-κB signaling has been recognized as one of the targets of thee phosphoinositide 3 kinase (PI3K)/protein kinase B (Akt) pathway, which can drive the aging process [21]. Accordingly, in this study, growing (G) and adult (A) rats were used to elucidate whether rice protein can exert an anti-inflammatory effect via suppression of the NF-κB pathway.

## 2. Results

### 2.1. Plasma ALT and AST Activities

**Figure 1:**
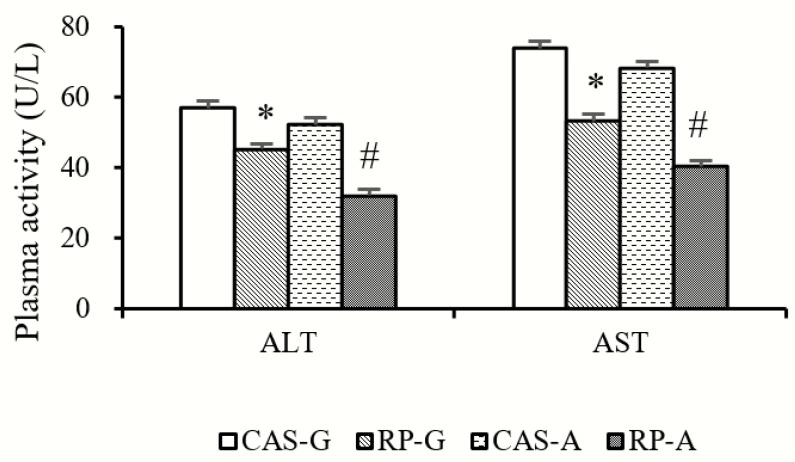
Plasma activities of ALT and AST. Values are the means ± SEM (*n* = 6). Bars marked with * are significantly different between CAS-G and RP-G (*p* < 0.05). Bars marked with # are significantly different between CAS-A and RP-A (*p* < 0.05). ALT, alanine transaminase; AST, aspartate transferase; CAS-A, adult rats fed with casein; CAS-G, growing rats fed with casein; RP-A, adult rats fed with rice protein; RP-G, growing rats fed with rice protein.

After 2 weeks feeding, compared with CAS-G (growing rats fed with casein) and CAS-A (adult rats fed with casein), the activities of alanine transaminase (ALT) (Figure 1) in plasma were significantly decreased by RP-G (growing rats fed with rice protein) to the degree of 20.47% in growing rats and decreased by RP-A (adult rats fed with rice protein) to the degree of 38.41% in adult rats (*p* < 0.05). Similarly, as shown in Figure 1, RP-G and RP-A significantly reduced plasma aspartate transaminase (AST) activities, accounting for a decrease of 27.77% in growing rats and decrease of 40.98% in adult rats (*p* < 0.05).

### 2.2. Hepatic NO Levels and iNOS Activity

**Figure 2:**
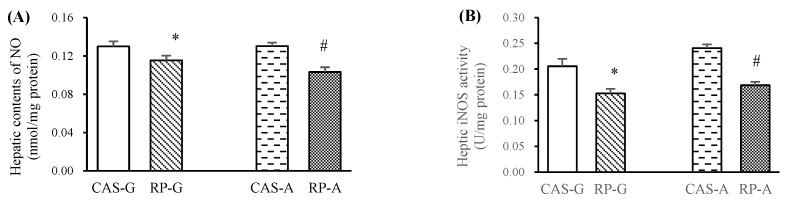
Hepatic contents of NO (**A**) and activities of iNOS (**B**). Values are the means ± SEM (*n* = 6). Bars marked with * are significantly different between CAS-G and RP-G (*p* < 0.05). Bars marked with # are significantly different between CAS-A and RP-A (*p* < 0.05). CAS-A, adult rats fed with casein; CAS-G, growing rats fed with casein; iNOS, inducible nitric oxide synthase; NO, nitric oxide; RP-A, adult rats fed with rice protein; RP-G, growing rats fed with rice protein.

Compared with CAS-G and CAS-A, RP-G and RP-A significantly reduced hepatic contents of NO in growing and adult rats (Figure 2A, *p* < 0.05). As illustrated in Figure 2B, RP-G and RP-A significantly reduced hepatic iNOS activities by 25.71% in growing rats and by 30.00% in adult rats (*p* < 0.05), further supporting the results that hepatic contents of NO could be reduced by rice protein feeding.

### 2.3. Hepatic ROS Accumulation

**Figure 3:**
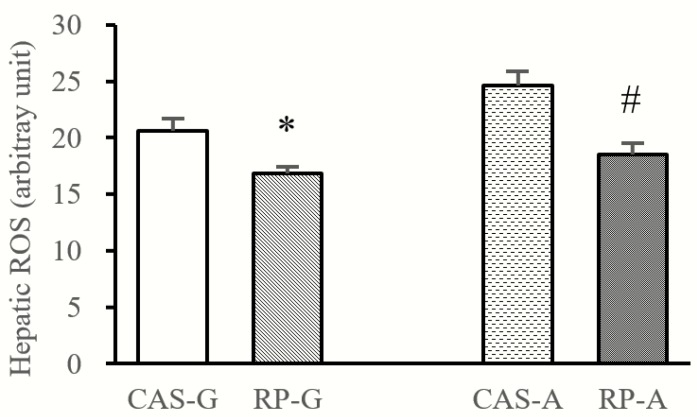
Hepatic contents of ROS. Values are the means ± SEM (*n* = 6). Bars marked with * are significantly different between CAS-G and RP-G (*p* < 0.05). Bars marked with # are significantly different between CAS-A and RP-A (*p* < 0.05). CAS-A, adult rats fed with casein; CAS-G, growing rats fed with casein; ROS, reactive oxygen species; RP-A, adult rats fed with rice protein; RP-G, growing rats fed with rice protein.

As shown in Figure 3, compared with CAS-G and CAS-A, hepatic contents of ROS were reduced by RP-G to the degree of 18.27% and reduced by RP-A to the degree of 24.71% (*p* < 0.05). The results suggest that hepatic ROS accumulation could be inhibited by rice protein.

### 2.4. Expressions of PI3K and AKT

**Figure 4:**
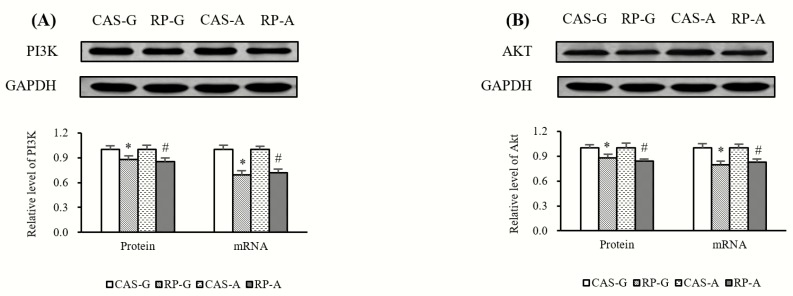
(**A**) Hepatic protein expression and mRNA levels of PI3K. (**B**) Hepatic protein expression and mRNA levels of Akt. Values are the means ± SEM (*n* = 6). Bars marked with * are significantly different between CAS-G and RP-G (*p* < 0.05). Bars marked with # are significantly different between CAS-A and RP-A (*p* < 0.05). AKT, protein kinase B; CAS-A, adult rats fed with casein; CAS-G, growing rats fed with casein; GAPDH, glyceraldehyde-3-phosphate dehydrogenase; PI3K, phosphoinositide 3 kinase; RP-A, adult rats fed with rice protein; RP-G, growing rats fed with rice protein.

Compared with CAS-G and CAS-A, the protein expression and mRNA levels of PI3K (Figure 4A) were significantly decreased by RP-G and RP-A in growing and adult rats (*p* < 0.05). Similarly, as illustrated in Figure 4B, RP-G and RP-A significantly reduced the protein expression and mRNA levels of Akt in growing and adult rats as compared to CAS-G and CAS-A (*p* < 0.05).

### 2.5. Expressions of NF-κB

**Figure 5:**
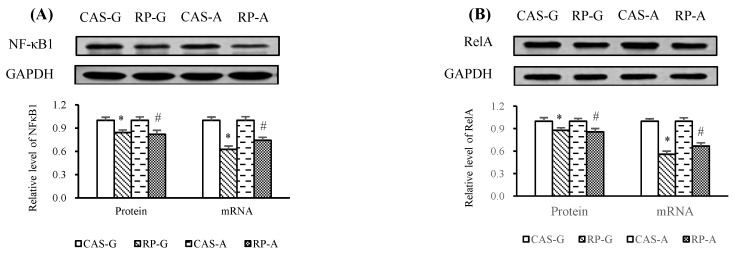
(**A**) Hepatic protein expression and mRNA levels of NF-κB1. (**B**) Hepatic protein expression and mRNA levels of RelA. Values are the means ± SEM (*n* = 6). Bars marked with * are significantly different between CAS-G and RP-G (*p* < 0.05). Bars marked with # are significantly different between CAS-A and RP-A (*p* < 0.05). CAS-A, adult rats fed with casein; CAS-G, growing rats fed with casein; GAPDH, glyceraldehyde-3-phosphate dehydrogenase; NF-κB1, nuclear factor-κB1; RelA, reticuloendotheliosis viral oncogene homolog A; RP-A, adult rats fed with rice protein; RP-G, growing rats fed with rice protein.

After 2 weeks of feeding, the inhibition of PI3K/Akt led to significantly decreased mRNA levels of NF-κB1 (Figure 5A) and RelA (Figure 5B) by RP-G and RP-A, with respect to CAS-G and CAS-A. Similarly, RP-G and RP-A downregulated the protein expression of NF-κB1 (Figure 5A) and RelA (Figure 5B) as compared to CAS-G and CAS-A, accounting for decreases of 15.72% (NF-κB1) and 12.11% (RelA) in growing rats, as well decreases of 18.01% (NF-κB1) and 14.17% (RelA) in adult rats (*p* < 0.05), respectively.

### 2.6. NF-κB Activation

In this study, the effects of rice protein on the activation of NF-κB were determined after 2 weeks feeding.

**Figure 6:**
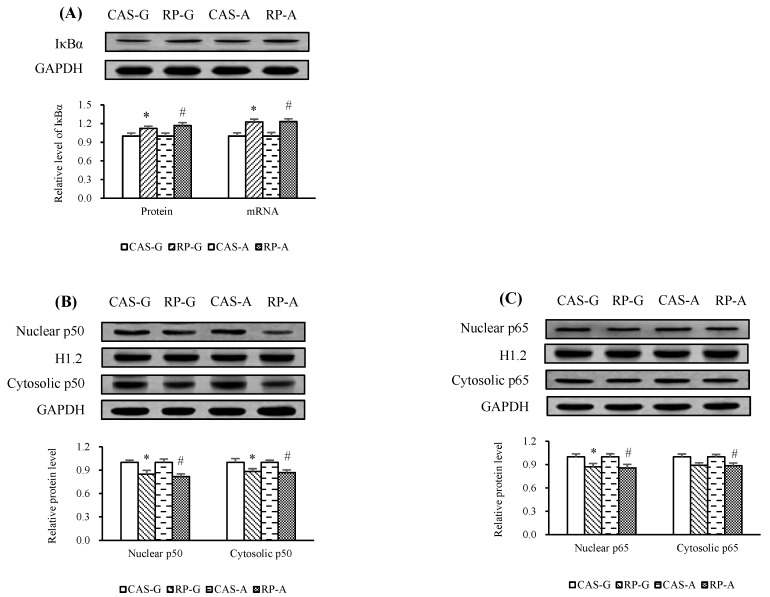
(**A**) Hepatic protein expression and mRNA levels of IκBα. (**B**) Nuclear and cytosolic protein contents of p50. (**C**) Nuclear and cytosolic protein contents of p65. Values are the means ± SEM (*n* = 6). Bars marked with * are significantly different between CAS-G and RP-G (*p* < 0.05). Bars marked with # are significantly different between CAS-A and RP-A (*p* < 0.05). CAS-A, adult rats fed with casein; CAS-G, growing rats fed with casein; GAPDH, glyceraldehyde-3-phosphate dehydrogenase; H1.2, histone cluster 1; IκBα, inhibitory κB α; p50, nuclear factor-κB 1; p65, reticuloendotheliosis viral oncogene homolog A; RP-A, adult rats fed with rice protein; RP-G, growing rats fed with rice protein.

Herein, we show that the inhibitory effect of rice protein in the expression levels of NF-κB can be attributed to the upregulation of both mRNA and protein levels of IκBα (Figure 6A). As a result, a decrease in the nuclear proportion of p50 was produced by RP-G to the degree of 15.02% in growing rats and by RP-A to the degree of 18.27% in adult rats (Figure 6B, *p* < 0.05). Similarly, RP-G and RP-A decreased the nuclear proportion of p65 by 12.86% in growing rats and by 14.26% in adult rats as compared to CAS-G and CAS-A (Figure 6C, *p* < 0.05). Also, RP-G and RP-A respectively reduced protein expression of cytosolic p50 (Figure 6B) and p65 (Figure 6C) as compared to CAS-G and CAS-A. These results suggest that rice protein could suppress NF-κB activation.

### 2.7. Expressions of Inflammatory Mediators

**Figure 7:**
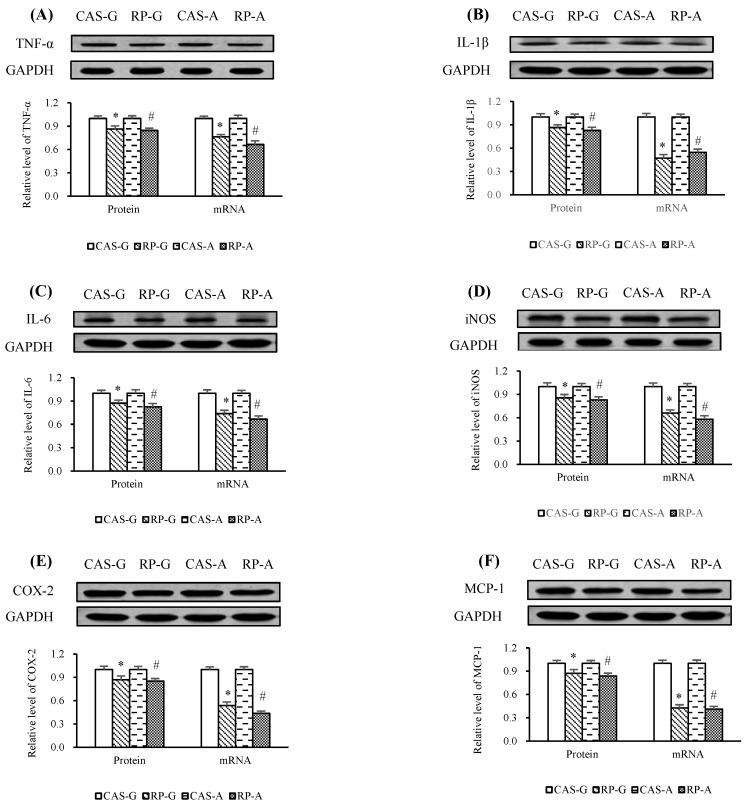
(**A**) Hepatic protein expression and mRNA levels of TNF-α. (**B**) Hepatic protein expression and mRNA levels of IL-1β. (**C**) Hepatic protein expression and mRNA levels of IL-6. (**D**) Hepatic protein expression and mRNA levels of iNOS. (**E**) Hepatic protein expression and mRNA levels of COX-2. (F) Hepatic protein expression and mRNA levels of MCP-1. Values are the means ± SEM (*n* = 6). Bars marked with * are significantly different between CAS-G and RP-G (*p* < 0.05). Bars marked with # are significantly different between CAS-A and RP-A (*p* < 0.05). CAS-A, adult rats fed with casein; CAS-G, growing rats fed with casein; COX-2, cyclooxygenase-2; GAPDH, glyceraldehyde-3-phosphate dehydrogenase; IL-1β, interleukin-1β; IL-6, interleukin-6; iNOS, inducible nitric oxide synthase; MCP-1, monocyte chemoattractant protein-1; RP-A, adult rats fed with rice protein; RP-G, growing rats fed with rice protein; TNF-α, tumor necrotic factor α.

After 2 weeks of feeding, the mRNA levels of TNF-α (Figure 7A), IL-1β (Figure 7B), IL-6 (Figure 7C), iNOS (Figure 7D), COX-2 (Figure 7E), and MCP-1 (Figure 7F), which are NF-κB-regulated inflammatory mediators, were markedly reduced by RP-G and RP-A, as compared to CAS-G and CAS-A (*p* < 0.05).

Compared with CAS-G and CAS-A, the protein expression of TNF-α (Figure 7A), IL-1β (Figure 7B), IL-6 (Figure 7C), iNOS (Figure 7D), COX-2 (Figure 7E), and MCP-1 (Figure 7F) were downregulated with the intake of rice protein, showing decreased protein levels from 13.61% (RP-G) to 15.47% (RP-A) in TNF-α (*p* < 0.05), from 13.61% (RP-G) to 17.24% (RP-A) in IL-1β (*p* < 0.05), from 12.80% (RP-G) to 17.48% (RP-A) in IL-6 (*p* < 0.05), from 14.43% (RP-G) to 17.21% (RP-A) in iNOS (*p* < 0.05), from 12.95% (RP-G) to 14.77% (RP-A) in COX-2 (*p* < 0.05), and from 12.58% (RP-G) to 16.24% (RP-A) in MCP-1 (*p* < 0.05), respectively. These results suggest that rice protein could prevent inflammation.

### 2.8. Expressions of Anti-Inflammatory Mediators

In addition to the downregulation of inflammatory mediators, after 2 weeks of feeding, the protein and gene expression of anti-inflammatory mediators, such as interleukin-10 (IL-10) and heme oxygenase 1 (HO-1), were also regulated by rice protein feeding.

**Figure 8:**
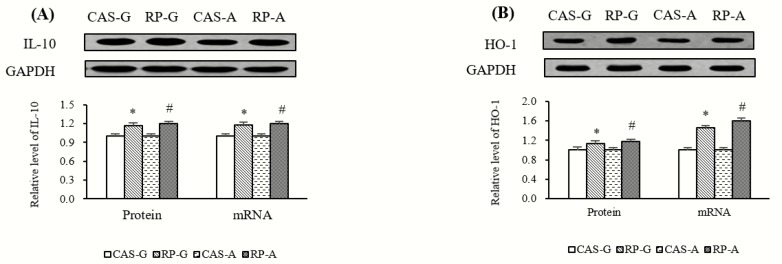
(**A**) Hepatic protein expressions and mRNA levels of IL-10. (**B**) Hepatic protein expressions and mRNA levels of HO-1. Values are the means ± SEM (*n* = 6). Bars marked with * are significantly different between CAS-G and RP-G (*p* < 0.05). Bars marked with # are significantly different between CAS-A and RP-A (*p* < 0.05). CAS-A, adult rats fed with casein; CAS-G, growing rats fed with casein; GAPDH, glyceraldehyde-3-phosphate dehydrogenase; HO-1, heme oxygenase 1; IL-10, interleukin-10; RP-A, adult rats fed with rice protein; RP-G, growing rats fed with rice protein.

As illustrated in Figure 8A, the mRNA levels and protein expression of IL-10 were significantly stimulated by rice protein feeding, accounting for enhancements of 18.26% (RP-G) to 20.05% (RP-A) in mRNA levels and 16.90% (RP-G) to 20.31% (RP-A) in protein expression with respect to CAS-G and CAS-A (*p* < 0.05). As shown in Figure 8B, compared with CAS-G and CAS-A, RP-G and RP-A significantly increased hepatic mRNA levels of HO-1 by 45.58% in growing rats and by 59.64% in adult rats (*p* < 0.05), while RP-G and RP-A upregulated the protein expression of HO-1 to the degree of 12.81% in growing rats and 17.26% in adult rats (*p* < 0.05). These results further supported the findings that rice protein could prevent inflammation.

## 3. Discussion

The present study demonstrates that rice protein can prevent inflammation in growing and adult rats. The anti-inflammatory effects of rice protein are attributable to suppression of the activation of NF-κB.

As a crucial regulator of chronic or acute inflammatory responses in inflammation, NO can be catalyzed via iNOS [1,22,23]. Thus, the anti-inflammatory effect is generally attributed to the decrease in NO production via suppression of iNOS activity and downregulation of iNOS expression. To support this view, in this study, the generations of NO were markedly reduced by RP-G and RP-A in growing and adult rats, reflecting that the status of inflammatory damage could be suppressed by rice proteins. These results were in agreement with our in vitro studies that rice protein could scavenge NO radicals [12,13]. Accordingly, hepatic iNOS activities and the expressions of iNOS were significantly reduced by RP-G and RP-A in growing and adult rats. On the other hand, as major hepatic inflammatory biomarkers, the activities of ALT and AST were significantly reduced by RP-G and RP-A, suggesting that rice protein could efficaciously improve hepatic inflammation in growing and adult rats. Taken together, in this study, it was evident that rice protein could inhibit inflammation in growing and adult rats.

The inflammatory process can be initiated by ROS-derived oxidative stress, suggesting that the overproduction of ROS may be a major contributor to the inflammation [1]. In light of this view, the anti-inflammatory response induced by rice protein should be focused on ROS-scavenging activity. In this study, the inhibitory effect of rice protein on hepatic ROS accumulation was observed in both growing and adult rats. These results were consistent with our previous findings that rice protein could scavenge ROS, including superoxide radical, hydrogen peroxide, etc. [11,12,24], which are the major cause of inflammation. In support, the findings observed in this study showed significant positive correlations between hepatic ROS content and ALT activity (r = 0.7888, *p* < 0.05), as well as AST activity (r = 0.8068, *p* < 0.05), suggesting that the decreased activity of ALT/AST was attributed to the reduced hepatic accumulation of ROS. Therefore, it is possible that the stronger ROS-scavenging capacity of rice protein might be closely linked with inhibition of the inflammatory process, further suggesting that rice protein can inhibit ROS-derived inflammation in growing and adult rats.

To elucidate the molecular mechanism by which rice protein induced an anti-inflammatory action, the influence of rice protein on the NF-κB pathway was assessed in this study. After 2 weeks of feeding, we could show that rice protein effectively and significantly inhibited NF-κB activation, although the activity of NF-κB was not directly determined in this study. It is clear that the activity of NF-κB can be primarily regulated by the interaction of IκB protein. Accordingly, the regulation of NF-κB-IκB interaction was particularly emphasized in this study as the key step for controlling NF-κB activity. As a master regulator of the inflammatory process, NF-κB is negatively regulated by IκB protein, e.g., IκBα. As stimulated by ROS-induced oxidative stress, IκBα is degraded and NF-κB is translocated into the nucleus and an inflammatory response can be induced. Subsequently, the inflammatory response is induced. Hence, NF-κB plays a crucial role in initiating inflammation [1,15]. With regard to this, the major finding in this study was that rice protein could enhance the expression of IκBα, suggesting that rice protein could augment the stabilization of the NF-κB-inhibitor. Consequently, the interaction of NF-κB-IκBα with rice protein was clearly observed in this study, implying that the activity of NF-κB could be suppressed by rice protein feeding. Supportably, RP-G and RP-A significantly inhibited the translocations of p50 and p65 into the nucleus, which were the major complexes of NF-κB. Results showed significant positive correlations between hepatic ROS and nuclear contents of NF-κB (p50, r = 0.8726, *p* < 0.05; p65, r = 0.9011, *p* < 0.05). Furthermore, it is clear that the canonical NF-κB pathway is characterized by activation of p50 and p65, suggesting that the inflammatory response is dependent on the nuclear translocations of p50 and p65 [15]. In this study, there were significant positive correlations between nuclear NF-κB and ALT (p50, r = 0.8100, *p* < 0.05; p65, r = 0.8107, *p* < 0.05), as well as AST (p50, r = 0.8397, *p* < 0.05; p65, r = 0.8478, *p* < 0.05), supporting the view that the downregulation of p50 and p65 could inhibit inflammatory action. In support of this notion, a significant negative correlation was observed between p50 and IL-10 (r = −0.8630, *p* < 0.05), as well as p65 and IL-10 (r = −0.8715, *p* < 0.05), in which IL-10 is an important anti-inflammatory mediator [25]. The present study, therefore, provides clear evidence that the suppression of NF-κB activation might be one of main anti-inflammatory mechanisms exerted by rice protein.

Upon suppression of NF-κB, downregulation of hepatic expressions in TNF-α, IL-1β, IL-6, iNOS, COX-2, and MCP-1, which are major inflammatory mediators regulated by NF-κB, were also observed in growing and adult rats fed with RP-G and RP-A. These results were consistent with an in vitro study reported by Wen et al., which indicated that rice protein hydrolysates inhibit the lipopolysaccharide-stimulated inflammatory response in RAW264.7 macrophages by inhibiting the release of NO and decreasing the expressions of TNF-α, iNOS, IL-6, and IL-1β, etc. [14]. More significantly, in this study, the results showed significant positive correlations between hepatic ROS and the expressions of TNF-α (r = 0.8858, *p* < 0.05), IL-1β (r = 0.9107, *p* < 0.05), IL-6 (r = 0.8465, *p* < 0.05), iNOS (r = 0.8620, *p* < 0.05), COX-2 (r = 0.8959, *p* < 0.05), and MCP-1 (r = 0.9235, *p* < 0.05). Thus, our findings further support the view that the ROS-induced inflammatory process could be prevented by rice protein in growing and adult rats.

Here, the question might arise of how rice protein can suppress NF-κB activation after 2 weeks of feeding. In order to identify the mechanism behind the rice protein-induced NF-κB inhibition, we investigated the PI3K/Akt signaling pathway in this study. NF-κB signaling has been recognized as one of the targets of the PI3K/Akt pathway [21,26]. Accordingly, the suppression of NF-κB activation might be a consequenc of downregulation of PI3K/Akt. To support this view, we found that RP-G and RP-A respectively decreased the gene and protein expressions of PI3K and Akt in growing and adult rats. More significantly, the results showed significant positive correlations between the expressions of PI3K/Akt and nuclear contents of p50 (PI3K, r = 0.8751, *p* < 0.05; Akt, r = 0.8491, *p* < 0.05), as well as nuclear p65 (PI3K, r = 0.9032, *p* < 0.05; Akt, r = 0.9477, *p* < 0.05). To support our findings, Madrid et al. suggested that Akt could stimulate the transactivation potential of the RelA/p65 subunit of NF-κB [27]. Therefore, it is not surprising that the downregulation of PI3K/Akt might be a switch to the suppression of NF-κB activation involving the anti-inflammatory action exerted by rice protein.

Now, another question might also emerge of why this anti-inflammatory response could be induced by rice protein. To explain this phenomenon, the view that the inflammation can be prevented by dietary antioxidants should be taken into account in this study [28]. It has been demonstrated that nuclear factor erythroid 2 (NF-E2)-related factor 2 (Nrf2) can modulate the anti-inflammatory response through the inhibition of NF-κB and upregulate antioxidant responsive element (ARE)-dependent gene expressions, such as HO-1 [11,29,30,31]. In light of this view, a satisfactory explanation could be drawn from our previous studies, in which rice protein could exert an antioxidant capacity via activation of Nrf2 and upregulation of HO-1 expression in adult rats [11]. Consistent with our previous studies, in this study, RP-G and RP-A effectively enhanced the expressions of HO-1, which is an important anti-inflammatory mediator, in growing and adult rats. Moreover, as another important anti-inflammatory mediator, the gene and protein expressions of IL-10 were also enhanced by RP-G and RP-A, further confirming the fact that rice protein could exert an anti-inflammatory effect. On the other hand, it has been demonstrated that the biological utilization of dietary protein is primarily dependent on its amino acid composition [7,8]. With regard to this, the fact that rice protein is rich in arginine particularly attracts our attention [5,9,10]. Some studies suggest that arginine can prevent or control the excessive inflammatory response through modulation of the immune system [32,33]. More importantly, our recent study showed that arginine could augment the expressions of HO-1 via activation of the Nrf2-ARE pathway [29]. In support, in this study, there were significant positive correlations between arginine intake and expressions of HO-1 (r = 0.9217, *p* < 0.05), as well as IL-10 (r = 0.8560, *p* < 0.05). Thus, the role of arginine in the stimulation of the anti-inflammatory response of rice protein should be emphasized in this study. The results showed a significant positive correlation between arginine intake and IκBα expression (r = 0.8620, *p* < 0.05), whereas there were the significantly negative correlations between arginine intake and expressions of PI3K (r = −0.8952, *p* < 0.05), as well as Akt (r = −0.9284, *p* < 0.05). Consequently, the significantly negative correlations between arginine consumption and nuclear contents of NF-κB (p50, r = −0.8765, *p* < 0.05; p65, r = −0.9471, *p* < 0.05) were clearly observed in this study. Thus, this study provides an insight that higher levels of arginine in rice protein (RP, 88.17 μg/mg; CAS, 33.33 μg/mg) might be a contributor to the anti-inflammatory effect of rice protein via suppression of NF-κB activation. In addition to arginine, rice protein is also rich in sulfur-containing amino acids (methionine and cystine), glycine, and glutamic acid, which could stimulate endogenous antioxidant activity to inhibit the inflammatory response.

NF-κB signaling is the molecular culprit of inflamm-aging, suggesting that the inflammatory process can be activated and accelerated with age [15,20]. However, under the present experimental condition, of interest was the finding that the increase of age did not cause a weakened anti-inflammatory capacity of rice protein in adult rats in comparison with growing rats. To explain this interesting phenomenon, the view that p53 and Nrf2 negatively regulates NF-κB signaling pathways in the aging process should be noted [34]. As longevity factors, both p53 and Nrf2 can protect inflamm-aging via inhibition of the NF-κB activation [21,34,35]. Recently, Liang et al. reported that rice protein could suppress DNA damage via activation of the ataxia-telangiectasia mutated (ATM)- checkpoint kinase 2 (Chk2)-p53 and Nrf2- Keap1 (Kelch-like ECH-associated protein 1) pathways in growing and adult rats [24], implying that rice protein might protect DNA damage-dependent inflamm-aging. Furthermore, in this study, increased age could not enhance the expressions of PI3K/Akt, which could drive the aging process [21], in adult rats fed with RP-A. Thus, in light of these facts, a satisfactory explanation that anti-inflammatory action via inhibition of NF-κB activation exerted by rice protein might be independent of age is convincing. Namely, this anti-inflammatory effect might be dependent on the amino acids profile of native rice protein, in which some amino acids, e.g., arginine, might play a key role in inducing the anti-inflammatory response to prevent inflammation. Clearly, additional studies are required to confirm this view.

## 4. Materials and Methods 

### 4.1. Protein Sources

Rice protein (RP) from *Oryza sativa* L. cv. *Longjing* 20 (Rice Research Institute of Heilongjiang Academy of Agricultural Sciences, Jiamusi, China) and casein (CAS) (Gansu Hualing Industrial Group, Gansu, China) were used as dietary protein sources in the present study.

### 4.2. Animals Experiments

According to our previous studies [6,7,8,11,24,29,30], animal studies (SCXK2012-0001, 8 February 2012) were approved and performed in conformity with the Guidelines of the Committee for the Experimental Animals of Harbin Institute of Technology (Harbin, China). Briefly, growing male Wistar rats (body weight 180–200 g) and adult male Wistar rats (body weight 390–410 g) were purchased from the Vital River Laboratories (Beijing Vital River Laboratory Animal Technology Co. Ltd., Beijing, China) and individually housed in metabolic cages in a room maintained at 22 ± 2 °C under a 12-h light–dark cycle (07:00–19:00 for light). In this study, four groups, consisting of six animals per group, were used for the investigation. Growing rats were respectively fed casein (CAS-G) and rice protein (RP-G) with a dietary protein level of 20% (as crude protein, CP) for 2 weeks, according to the formula recommended by American Institute of Nutrition for growth (AIN-93G) [36]. Adult rats were respectively fed 14% (as CP) dietary proteins of casein (CAS-A) and rice protein (RP-A) for 2 weeks, according to the formula recommended by American Institute of Nutrition for adult maintenance (AIN-93M) [36].

### 4.3. Measurement of Plasma Enzyme Activity

The activities of plasma ALT and AST, the major inflammatory biomarkers, were determined using the methods described in the kits from Nanjing Jiancheng Bioengineering Institute (Nanjing, China).

### 4.4. Analyses of Hepatic NO Level and iNOS Activity

The contents of NO in the liver and hepatic activity of iNOS were measured using the commercial kits (Nanjing Jiancheng Bioengineering Institute, Nanjing, China).

### 4.5. Determination of Hepatic ROS

The analysis of ROS levels in the liver was according to our previous studies [11,24,29,30]. Briefly, ROS was determined by fluorescence of 2′, 7′-dichlorofluorescin diacetate (DCF-DA) as described by the manufacturer’s protocol of the commercial kit (Nanjing Jiancheng Bioengineering Institute, Nanjing, China). The fluorescence intensity was measured at the 485-nm excitation wavelength and 530-nm emission wavelength. Data are expressed as an arbitrary unit of fluorescent intensity per µg protein.

### 4.6. Quantitative Real-Time PCR

Quantitative real-time PCR was measured in this study, according to our previous studies [24,29,30]. Briefly, using the TRIzol reagent kit (Invitrogen, Carlsbad, CA, USA) and the PrimeScript™ 1st strand cDNA Synthesis Kit (Takara Bio. Inc., Otsu, Shiga, Japan), total RNA was extracted from the livers and cDNA was reverse transcribed. The mRNA level of GAPDH (glyceraldehyde-3-phosphate dehydrogenase) was treated as a normalization. The primers sequences used are shown in Table 1. In this study, the relative mRNA level in group CAS-G and CAS-A was respectively set as 1.00.

### 4.7. Western Blotting Analysis

The total, cytoplasmic, and nuclear proteins were used for western blot analysis, which were prepared as described in our previous studies [24,29,30]. In brief, primary antibodies for IκBα (Proteintech, Wuhan, China), NF-κB1 (p105/p50, Proteintech), RelA (p65, Proteintech), iNOS (Proteintech), IL-1β (Proteintech), IL-6 (Proteintech), TNF-α (Proteintech), COX-2 (Proteintech), MCP-1 (Abcam, Cambridge, UK), PI3K (Proteintech), Akt (Proteintech), IL-10 (Santa Cruz Biotechnology, Santa Cruz, CA, USA), HO-1 (Proteintech), H1.2 (Proteintech), and GAPDH (Proteintech) were used in this study. The second antibody were purchased from Santa Cruz Biotechnology. In this study, the relative protein expression in group CAS-G and CAS-A was respectively set as 1.00.

### 4.8. Statistical Analysis

The statistical analysis was in accordance with our previous studies [6,7,8,11,24,29,30]. Briefly, data are expressed as the mean ± SEM. Differences between groups were examined for statistical significance using one-way analysis of variance (ANOVA) followed by the least significant difference test. The criterion for significance was *p* < 0.05.

## 5. Conclusions

**Figure 9:**
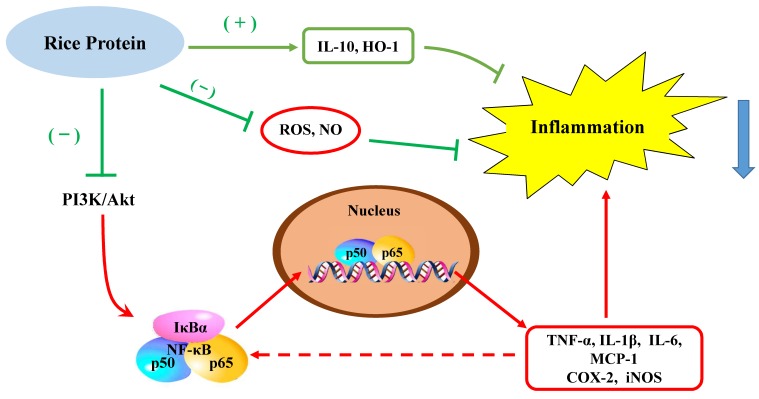
Rice protein exerts an anti-inflammatory effect via suppression of the NF-κB pathway. The green “T” represents for the inhibitory interaction; the red arrow shows the up-regulatory effect; the blue arrow indicates that rice protein can suppress the inflammatory action; the “(+)” represents the activation; the “(−)” represents the inhibition. Akt, protein kinase B; COX-2, cyclooxygenase-2; HO-1, heme oxygenase-1; IκBα, inhibitory κB α; IL-1β, interleukin-1β; IL-6, interleukin-6; IL-10, interleukin-1β; iNOS, inducible nitric oxide synthase; MCP-1, monocyte chemoattractant protein- 1; NF-κB, nuclear factor-κB; NO, nitric oxide; PI3K, phosphoinositide 3 kinase; p50, nuclear factor-κB1; p65, reticuloendotheliosis viral oncogene homolog A; ROS, reactive oxygen species; TNF-α, tumor necrotic factor α.

The present study is the first to demonstrate that rice protein exerts anti-inflammatory effects in growing and adult rats. The results indicate that the anti-inflammatory response induced by rice protein is primarily attributed to suppression of the NF-κB pathway (Figure 9). Significantly, the study confirms a link between the inhibition of ROS-derived inflammation with the intake of rice protein in growing and adult rats. The novel finding observed in this study is that the anti-inflammatory activity of rice protein cannot be attenuated by increased age. Clearly, more detailed investigations are needed to explore the precise anti-inflammatory mechanisms exerted by rice protein in further study.

## Figures and Tables

**Table 1:** Sequences of primers for quantitative real-time PCR.

Gene	Forward	Reverse
*GAPDH*	ACAGCAACAGGGTGGTGGAC	TTTGAGGGTGCAGCGAACTT
*NF-κB1*	TATGGGCAGGATGGACCTA	TCAGAGCCAAGAAAGGAAGC
*RelA*	TGTGAACCAATTCGCCGAGAAGG	CTCAGCCAGCCAGTGCTTGTC
*IκBα*	GAAGGACGAGGATTACGAGCAGATG	ATGGTCAGTGTCTTCTCTTCATGGATG
*Akt*	ACTCATTCCAGACCCACGAC	AGCCCGAAGTCCGTTATCTT
*PI3K*	TATTGCGAGGGAAACGAGAT	CCAGGGAGGTGTGTTGGTAA
*TNF-α*	TGCCTCAGCCTCTTCTCATT	GCTTGGTGGTTTGCTACGAC
*IL-1β*	TCACAGCAGCATCTCGACAA	GGTCCTCATCCTGGAAGCTC
*IL-6*	TCCGTTTCTACCTGGAGTTTG	GTTGGATGGTCTTGGTCCTT
*iNOS*	GATGTGCTGCCTCTGGTCCT	GAGCTCCTGGAACCACTCGT
*COX-2*	AGCGACTGTTCCAAACCAGC	CCTCTTGGCGAGGGAGATGG
*MCP-1*	GGCCTGTTGTTCACAGTTGC	GTTCTCCAGCCGACTCATTG
*IL-10*	GCACTGCTATGTTGCCTGCT	TCAGCTCTCGGAGCATGTG
*HO-1*	GCCCTGGAAGAGGAGATAGAG	TAGTGCTGTGTGGCTGGTGT
